# A Hybrid Artificial Neural Network Approach for Modeling the Behavior of Polyethylene Terephthalate (PET) Under Conditions Applicable to Stretch Blow Molding

**DOI:** 10.3390/polym17081067

**Published:** 2025-04-15

**Authors:** Fei Teng, Gary Menary, Shiyong Yan, James Nixon, John Boyet Stevens

**Affiliations:** 1 Sinopec Research Institute of Petroleum Processing Co., Ltd., Beijing 100083, China; 2 School of Mechanical and Aerospace Engineering, Queen’s University Belfast, Belfast BT7 1NN, UK; shiyong@bmt-ni.com (S.Y.); james@bmt-ni.com (J.N.); 3 The Procter & Gamble Company, Corporate R&D, Cincinnati, OH 45202, USA; stevens.jb.2@pg.com

**Keywords:** numerical algorithms, multiaxial stress–strain behavior, artificial neural network (ANN), rate-dependent material, stretch blow molding

## Abstract

Stretch blow molding (SBM) is widely utilized in industrial applications, yet the deformation characteristics of materials during this process are intricate and challenging to precisely articulate. To accurately forecast the stress–strain response of polyethylene terephthalate (PET) in SBM, a hybrid Artificial Neural Network (ANN)-based constitutive model has been developed. The model has been created by combining a physical-based function for capturing the small-strain behavior in parallel with an ANN-based model for capturing the temperature-dependent large-strain nonlinear viscoelastic behavior. The architecture of the ANN has been designed to ensure stability in a load-controlled scenario, thus making it suitable for use in FEA simulations of stretch blow molding. Data for training the model have been generated by a new semi-automatic experimental rig which is able to produce 850 stress–strain curves over a wide range of process conditions (temperature range 95–115 °C and strain rates ranging from 1/s to 100/s) directly from blowing preforms using a combination of high-speed video, digital image correlation and sensors for pressure and force. The model has already been implemented in the commercial FEA package Abaqus via a VUMAT subroutine, with its performance validated by comparing the prediction of the evolution of preform shape during blowing vs. high-speed images. In conclusion, the developed hybrid ANN model, when integrated into Abaqus, offers a more accurate simulation of SBM processes, contributing to improved design efficiency and product quality.

## 1. Introduction

**Figure 1:**
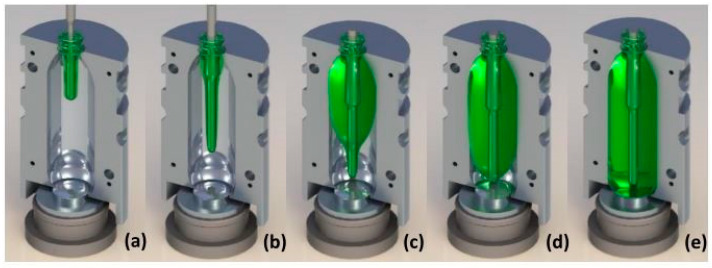
The forming stages in the SBM process include (**a**) the beginning of SBM; (**b**) the preform being stretched by the stretch rod; (**c**) low-pressure air being introduced into the preform; (**d**) high-pressure air continuously entering the preform; (**e**) the completion of the SBM process.

Stretch blow molding (SBM) is the most common method for producing polyethylene terephthalate (PET) bottles used in the carbonated soft drink (CSD) water and consumer product industries. The first step involves creating a preform, which is a test tube-shaped specimen made through injection molding. The preform is then heated above its glass transition temperature and shaped into a mold using both axial stretching through a stretch rod and radial stretching via internal air pressure. The typical evolution of the preform as it is stretched and blown in the mold is shown in Figure 1. Figure 1a depicts the beginning of SBM, Figure 1b represents the moment when the stretch rod is in touch with the preform, Figure 1c shows the geometry of the preform as low pressure air (~5 bar) is introduced (known as preblow). Figure 1d,e represent the introduction of high pressure air (~>30 bar) know as final blow which forms the details of the bottle.

Manufacturers face a significant challenge in creating containers that utilize minimal material while still meeting the performance demands of in-service use. Traditionally, the optimum preform design and process conditions have been determined by trial and error and knowhow; however, the more modern approach involves the use of simulation whereby a finite element model of SBM is used to evaluate preform design and process conditions in advance. One of the key inputs into the SBM simulation is the material model that is required to accurately capture the nonlinear viscoelastic behavior of PET subjected to high deformation and high strain rates at temperatures above the glass transition temperature (T_g_). Previous work by the authors has demonstrated that a model originally developed by Buckley et al. [1,2,3] and with subsequent adjustments [4,5,6] can reasonably well capture this complex behavior within SBM simulations. However, the model has a limitation, namely the complexity of generating the material parameters, which can be time-consuming and expensive, and it can have difficulty maintaining accuracy across a wide range of temperature and strain rates.

For decades, researchers have been trying to develop accurate physical-based models which are able to describe the nonlinear viscoelastic behavior of PET during the SBM process at temperatures above the glass transition temperature (T_g_). A comprehensive review of these approaches has been presented in our previous publication [7,8]. In summary, all of these approaches can capture the typical nonlinear temperature stress–strain behavior in specific conditions at different levels of accuracy. However, despite efforts to develop accurate models, weaknesses still exist due to the highly nonlinear deformation behavior of PET during blow molding, which is affected by various parameters including temperature, deformation mode, stretch ratio, strain history, and strain rate. As pointed out by Menary et al. [9], these parameters have a significant impact on the deformation behavior of PET. A shared characteristic among these constitutive models mentioned above is that they limit the depiction of the material’s deformation behavior to predefined parametric constitutive equations. However, fixed equations fail to accurately match the complexity of real conditions, as such physical models have associated inaccuracies.

According to Liu et al. [10], the most obvious novelties of the ANN-based constitutive model is that it is able to build a complex nonlinear relationship in a form-free manner. As a result, the weakness of these models that depend on presumed functions with fixed mathematic expressions can be navigated by utilizing ANN technology. In addition to the ‘form-free’ advantage mentioned above, the ANN also has other unique advantages compared with conventional physical models. Zhang et al. [11] demonstrated that a single neural network model was able to directly describe the behavior of a material during deformation without any other equations, such as yield function and hardening law. As a result, in comparison with a physical-based model, the complexity of a constitutive model can be greatly reduced, which brings the dual advantage of reduced running time and coding simplicity.

One of the first studies to propose the concept of an ANN-based constitutive model was conducted by Ghaboussi et al. [12]. They utilized an ANN-based constitutive model to forecast the stress–strain correlation of concrete when subjected to biaxial monotonic loading and uniaxial cyclic loading. Thanks to advancements in computer power, ANN-based constitutive models have undergone rapid development in recent years and have already found widespread use in various materials, such as foam [13], metals [14,15,16,17], and polymers [18,19,20].

Settgast et al. [13] proposed a hybrid ANN-based model that embedded neural networks into the established framework of rate-independent plasticity and was able to simulate the elastic–plastic behavior of foam efficiently. Settgast et al. [13] proved the fact that not only can the ANN function be used by itself but also that there were advantages in combining it with an existing traditional physical constitutive model. The cooperation with physical-based functions not only reduced the size of the training database required for training the ANN but also improved its stability.

Pandya et al. [14] upgraded Kessler’s model [21] and proposed a machine learning-based plasticity model which took strain rate and temperature into account. Li et al. [15,16] proposed an ANN to capture the large deformation response of the DP780 steel over a large range of strain rates and temperature. This ANN-based model not only has higher accuracy and significant speed-up compared with the conventional plasticity model but also features a hardening law. Although Li and colleagues’ previous work was in the metal domain, they demonstrated the feasibility of replacing conventional physical constitutive models with ANNs.

Diamantopoulou et al. [18] trained an ANN to describe the relationship among the process parameters, the degree of polymerization, and the nonlinear stress–strain response of a polymer structure obtained from experiments. They highlighted the robustness of the developed ANN model and its advantage of reducing the complexity of the constitutive law. The hybrid modeling approach proposed by Jordan et al. [19] combines a mechanism-based model and a data-based model. However, their attention is limited to the stress–strain response for uniaxial experiments only, and their temperature and strain rate range are relatively small (20–80 °C and 10^−3^/s–10^−1^/s) compared to the strain rates and temperature range required for modeling PET in SBM (1/s–100/s and 85–115 °C). Jang et al. [20] proposed a different combination method involving FEA and an ANN in that the ANN was only used to replace the nonlinear iterations in the stress return mapping method within a UMAT user subroutine [22].

The aim of this paper is to develop an ANN-based constitutive model to predict the complex behavior of PET in SBM. In order to do this, the authors will also present an experimental method for efficiently generating the rich data set required for producing a robust and accurate ANN model (850 strain–stress curves). Each strain–stress curve consists of more than 200 data points so that 253,864 data points can be collected during experiments. This study builds on previous publications [7,8] where the authors demonstrated the ability of an ANN to predict the behavior of PET biaxially stretched at different strain rates and temperatures for a simple displacement-controlled planar experiment. In this paper, the authors have increased the complexity and capability of the ANN model though the development of a new architecture enabling it to be used in the commercial finite element package Abaqus for complex load-controlled scenarios suitable for modeling the behavior of PET in SBM.

The novelty of the current work can be summarized into two points. Firstly, an important point to note is that most of the ANN models discussed have been validated for displacement-controlled scenarios, i.e., the boundary conditions for displacement are imposed on the model and the resulting force is calculated. However, to be used in a complex manufacturing simulation such as SBM, it is also important to validate the model in a load-controlled scenario, i.e., a load is applied and the corresponding displacement is calculated. In order to deal with this difficulty, a hybrid constitutive model combining advantages from both a conventional physical-based model and an ANN-based model is proposed in the current work. The ANN part in the hybrid constitutive model adopts an innovative architecture specifically designed for load-controlled scenarios.

Another novelty of the current work is the acquisition system of experimental data. Generally, the experimental data required to train an ANN are acquired from standard specimens with simple graphs; for example, Jordan et al. [19] utilized dog bone specimens. However, as highlighted by Menary et al. [9], the SBM process is a complex manufacturing process influenced by many dynamic parameters. As a result, in order to obtain an accurate enough ANN model, experimental data were collected from free stretch blow (FSB) experiments directly which are able to imitate real process parameters. All experimental data shown in the current work are published for the first time.

This paper is structured as follows. Section 2 describes the experimental procedure for generating the rich experimental data set necessary for training the ANN model. The component of the conventional physical-based model used is described in Section 3, whilst Section 4 is utilized to introduce the detailed architecture of the hybrid ANN constitutive model. Subsequently, the hybrid ANN-based model is trained in Section 5. Finally, in Section 6, the hybrid model is implemented in the finite element package Abaqus to simulate the behavior of PET in a SBM simulation with the results validated against the experimental data.

## 2. Experimental Procedure

### 2.1. Material and Specimens

**Figure 2:**
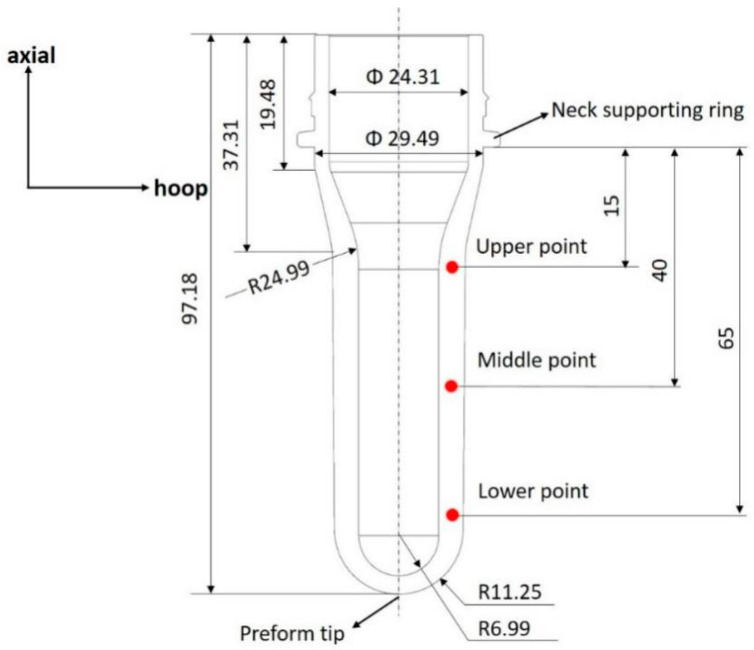
Dimensions of preform used in experiments.

The supplied PET material has an intrinsic viscosity of 0.81 ± 0.02 dL/g and a density of 1.33 g/cm^3^ and was provided by DAK Americas for use in the current work. Further details on the material are available in a previous work by the authors [7,8]. The drawing of the preform is shown in Figure 2 with all dimensions in millimeters. The bottle design dimensions are provided by Procter & Gamble and represent one of the preform sizes utilized in industrial manufacturing. Consequently, this study also employs preforms of identical dimensions for experimentation.

### 2.2. Free Stretch Blow Test

**Figure 3:**
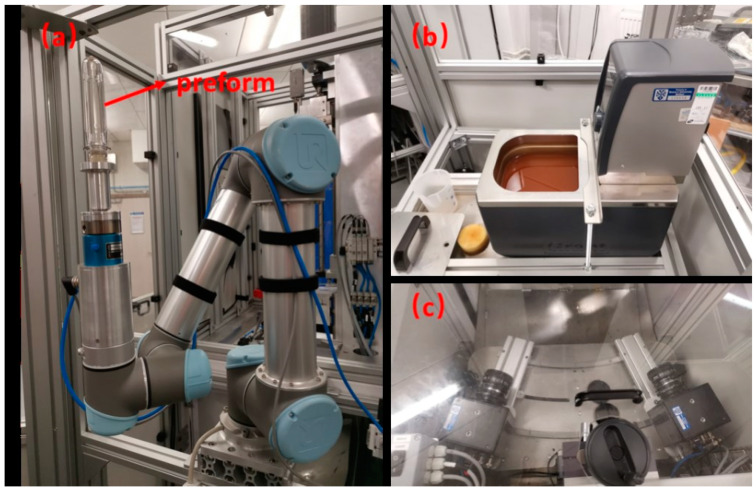
The robotic controlled test rig: (**a**) the robotic arm used to hold the specimen; (**b**) the oil bath device; (**c**) high-speed cameras used for monitoring the deformation.

A new robotic controlled test rig (Figure 3) has been developed by the authors which is capable of automatically capturing stress–strain data directly from a blowing preform as a function of process conditions (temperature, pressure, mass flow rate of air).

The test method consists of applying a pattern to a preform that is heated in an oil bath to provide uniform temperature (Figure 3b) and subsequently blown without the constraint of a mold, whilst the evolving preform shape and pattern evolution are recorded by two high-speed cameras (Figure 3c). The movement between the different stages is controlled by a robot (Universal R5) (Figure 3a), and hence, there is potential for automation.

**Figure 4:**
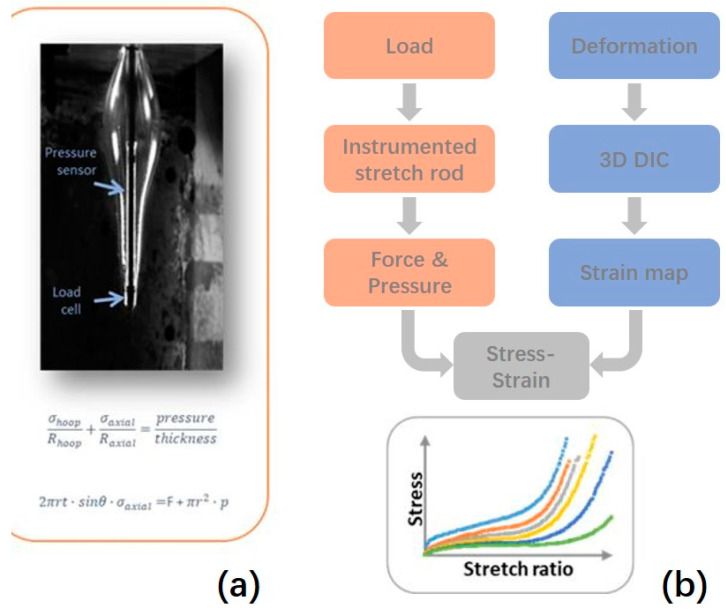
Schematic diagram of demonstrating how stress–strain data are captured from free stretch blow experiments: (**a**) schematic diagram of stretch rod; (**b**) stress–strain diagram.

The rig is instrumented with a pressure sensor and load cell in the stretch rod [23] which records the internal pressure and axial force on the stretch rod over time as the preform is blown (Figure 4a). Digital image correlation (DIC) then produces a full-field strain map of the deforming surface of the preform whilst the pressure and force data can be converted to stress using thin-walled membrane theory [24]. With the help of the stretch rod device and the 3D DIC technology, the stress–strain/stretch ratio data for every point on the preform can be calculated (Figure 4b).

**Figure 5:**
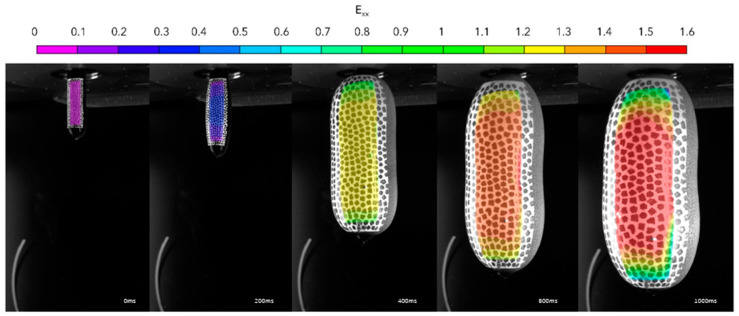
Example of evolution of blowing preform monitored by high-speed camera with contours of hoop strain as calculated by digital image correlation.

**Figure 6:**
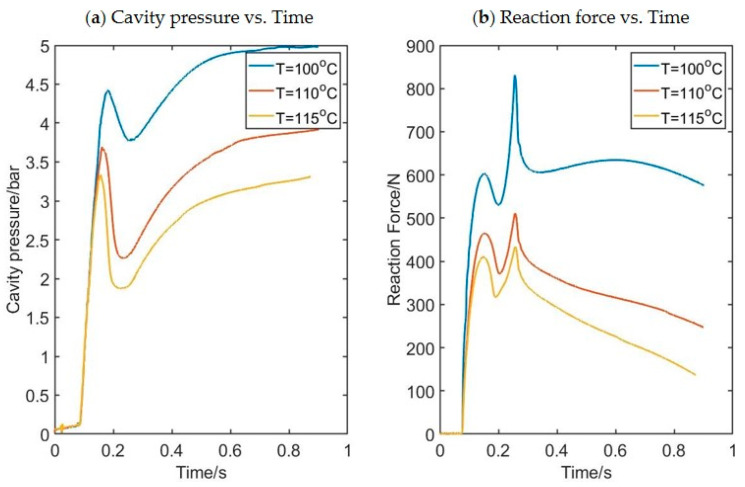
The evolution of force and pressure in the experiment shown in Figure 5.

Figure 5 shows a typical image of the blowing preform as recorded by the high-speed cameras with the contours representing the hoop strain, whilst Figure 6 shows the influence of temperature on the evolution of force and pressure for a preform recorded by the pressure sensor and load cell shown in Figure 4a.

**Figure 7:**
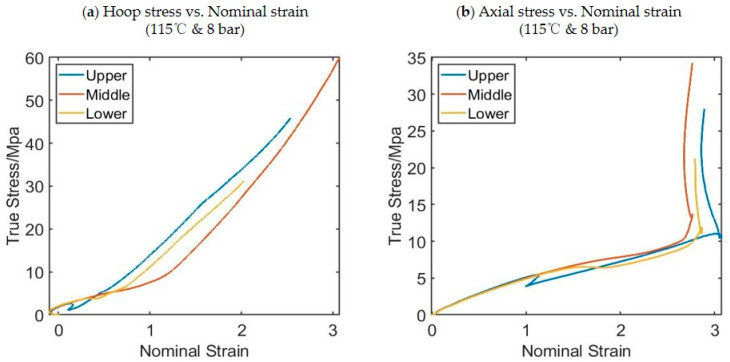
An example of hoop stress strain curves (**a**) and axial stress strain curves (**b**) from three positions of the preform (upper, middle, and lower as indicated in Figure 2).

**Figure 8:**
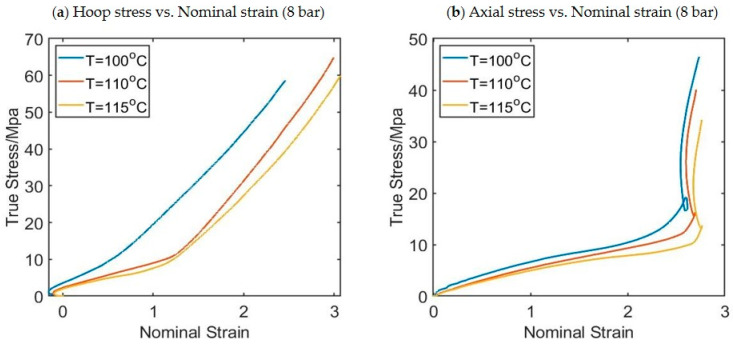
An example of hoop stress strain curves (**a**) and axial stress strain curves (**b**) from the midpoint of the preform at three different temperatures.

Stress–strain curves are available for every pixel in the DIC analysis, providing hundreds of stress strain curves for each process condition. In each free-flow experiment, 85 strain–stress curves are selected from the experiments corresponding to different pixels and they will be used as a training database for the model to be developed. Figure 7 shows an example of the stress–strain curves produced at three different locations along the length of the preform for a preform blown at 115 °C and a pressure of 8 bar, thus highlighting the influence of strain history/deformation mode on material behavior. The three locations are known as the upper point, middle point, and lower point. The three positions on the preform are defined in Figure 2. An example of stress–strain curves in both the hoop direction (a) and axial direction (b) taken from the midpoint of the preform for different preform temperatures is shown in Figure 8. It is worth noting that all experiments were repeated three times and the values of stress and strain are the averages of these three experimental values.

The rig is ideal for efficiently producing a rich data set covering a wide range of process conditions directly relevant to SBM and is therefore suitable for producing data that can subsequently be used to train an Artificial Neural Network (ANN) to model its behavior.

As a typical viscoelastic material, PET’s mechanical properties during the SBM process are primarily influenced by temperature, stretch rate, and strain history. Temperature and stretch rate are more readily controllable, while strain history is adjusted indirectly by varying these parameters. During the SBM process, the stretch rate is regulated by controlling the mass flow rate of air entering the preform. As a result, a series of experiments were carried out by using a mixed-level full factorial design of experiments (DoE) with two factors—temperature and mass flow rate of air entering the preform. Due to its known influence on behavior, the temperature was discretized into five levels ranging from 95 °C (just above the glass transition temperature) to 115 °C (just below the cold crystallization temperature). The mass flow rate was discretized into two levels (low and high). As a result, there are 10 different experiments (also known as 10 DoEs) involved in the current paper, as shown in Table 1. Each experiment (DoE) was repeated three times. The average values of stress and strain obtained from these three experiments were treated as experimental values of this DoE and were used to train the model. As mentioned above, 85 strain–stress curves were selected from one free stretch blow experiment corresponding to different heights along the preform, and our experiment equipment was able to record stress and strain values every 0.0005 s during the free stretch blow experiments.

**Figure 9:**
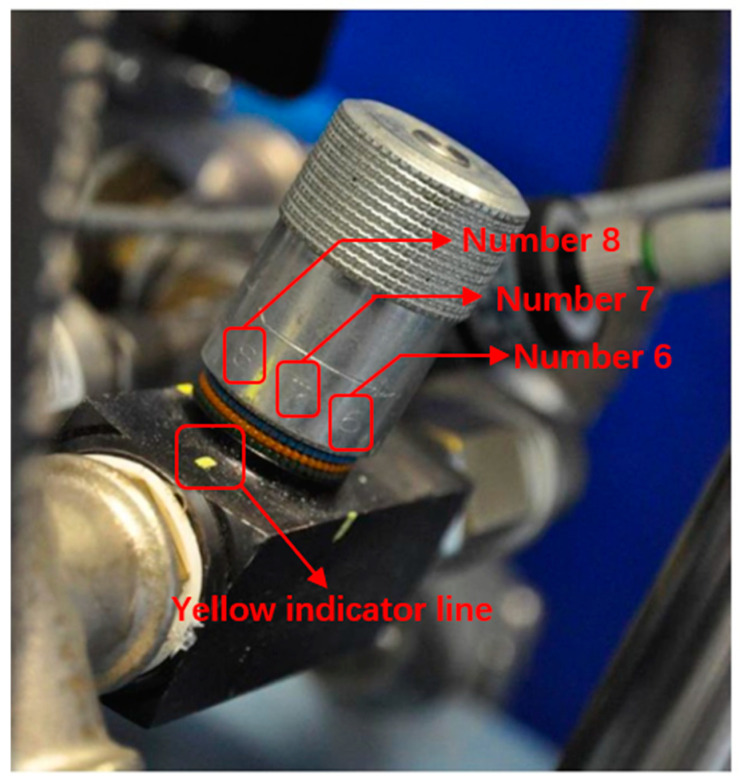
A flow restrictor valve with a rotating knob.

The mass flow rate of air was controlled by adjusting a flow restrictor valve, as shown in Figure 9. The flow restrictor valve has numbers engraved at its base, which, in conjunction with the yellow indicator line at the bottom, are used to quantitatively describe the degree of opening and closing of the flow restrictor valve.

A high mass flow rate means that the rotating knob is set to a flow index of ‘6’; in other words, the position engraved with the number 6 is aligned with the yellow indicator line. Conversely, a low mass flow rate corresponds to the flow index ‘2’ on the rotating knob, which indicates ‘nearly closed’. Based on the calculation of Salomeia et al. [25], the low-flow setting corresponds to a mass flow rate of 8.88 ± 0.195 g/s, whilst the high flow rate corresponds to a mass flow rate of 33.96 ± 0.863 g/s. The flow rate of air effectively controls the rate of inflation of the preform and hence influences the strain rate of the deforming material. The stretch rod displacement and velocity were set at fixed values of 130 mm and 0.5 m/sec, respectively, for all experiments, whilst the valve for enabling the air to enter the preform was opened at the same time when the stretch rod touched the tip of the preform (known within the bottle blowning industry as P0).

Further details on the experimental setup are presented in the thesis of Yan [4]. A summary of all of the experiments conducted is shown in Table 1. In Table 1, P represents pressure, N is the flow index of the flow restrictor valve, and T is the temperature.

## 3. Brief Description of Buckley Model

**Figure 10:**
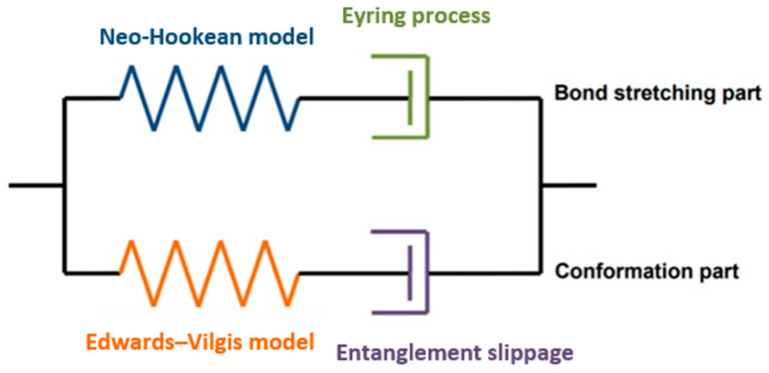
Spring–dashpot illustration of Buckley model.

Menary et al. [26] compared several physical-based models, including a hyperelastic model, a creep model, and the Buckley model, to ascertain their suitability for modeling stretch blow molding. The model proposed by Buckley et al. [1,2,3] was found to provide the simulation results that match best with the experimental data. After Menary’s work, the model has evolved to capture key features of the behavior of PET including how the onset of strain hardening changed as a function of temperature and strain rate [4,5,27]. A 1D representation of the Buckley model is shown in Figure 10 which shows a parallel circuit with two arms, named the bond stretching part and the conformation part.

The bond stretch arm represents the bond deformation of the polymer, exhibiting the instantaneous stress from the interactions of the molecular bonds and is important for predicting the onset of yield and how it varies with temperature and strain rate. In the conformational arm, it represents the perturbation of the polymers’ conformation and is determined by the change in an entropic free energy function. The conformation part captures the large-strain behavior post yield including strain softening and strain hardening and how these change as a function of temperature, strain rate, and mode of deformation.

The Buckley model can be expressed by Equation (1),

(1) $$\sigma_{i}=\sigma_{i}^{b}+\sigma_{i}^{c}(i=1,2,3),$$

where $\sigma_{i}^{b}$ is the principal bond stretching stress and $\sigma_{i}^{c}$ is the principal conformation stress.

### 3.1. Bond Stretching Part

The principal deviatoric stress of the bond stretching component ($S^{b}$) is expressed by Equation (2),

(2) 2Gbdedt=dSbdt+Sbτ,

where $G^{b}$ is the contribution to shear modulus arising from bond stretching, τ is the relaxation time, and e is the deviatoric true strain. In the present work, $G^{b}$ is set as 600 MPa [1]. In order to obtain τ, three equations are utilized, including the Eyring formulation [28], the Vogel–Tammann–Fulcher function [29,30], and the Arrhenius equation [31].

According to Li & Buckley [32], an implicit method or identified integral solution for Equation (2) can cause potential numerical difficulty in modeling large and post-yield deformations. In order to solve this problem, an explicit method was proposed to solve $S^{b}$ from Equation (2), as shown in Equations (3) and (4), which have been subsequently used in the VUMAT subroutine developed in the current work.

(3) $$S_{t+\Delta t}^{b}=S_{t}^{b}+\Delta S_{t+\Delta t}^{b}+(WS_{t}^{b}-S_{t}^{b}W)\Delta t$$

Δt is the time increment, W is the spin tensor, and $\Delta S_{t+\Delta t}^{b}$ is the stress increment in this time increment. $\Delta S_{t+\Delta t}^{b}$ is obtained from Equation (4).

(4) ΔSt+Δtb=1−exp−Δtτt2Gbdedtτt−Stb

### 3.2. Conformation Part

In the conformational part, the stress component ($S^{c}$) is represented by the crosslinks–sliplinks model proposed by Edwards and Vilgis [33]. The principle conformational stresses ($S_{i}^{c}$) are expressed by Equation (5),

(5) Sic=λiJ∂Ac∂λi−p,

where p is the hydrostatic stress, J is the determinant of the deformation gradient tensor, $\lambda_{i}$ is the principal stretch, and $A^{c}$ is obtained from the free energy function which was derived by Edwards and Vilgis [33] as shown in Equation (6):

(6) $$A^{C}=\frac{N_{e}k_{B}T}{2}\left[\frac{\left(1+\eta \right)\left(1-\alpha^{2}\right)}{1-\alpha^{2}\sum_{i=1}^{3}{\lambda_{i}^{n}}^{2}}\sum_{i=1}^{3}\frac{{\lambda_{i}^{n}}^{2}}{1+{{\eta \lambda }_{i}^{n}}^{2}}+\sum_{i=1}^{3}ln\left(1+{{\eta \lambda }_{i}^{n}}^{2}\right)\right.\;\left.+ln(1-\alpha^{2}\sum_{i=1}^{3}{\lambda_{i}^{n}}^{2})\right]$$

where $N_{e}$ is the entanglement density, $k_{B}$ is the Boltzmann’s constant, T is the absolute temperature, η is the looseness parameter of the entanglement, $\lambda_{i}^{n}$ is the principal network stretch, and α is a measurement of the inextensibility of the entanglement network where the maximum extension is determined by $\frac{1}{\alpha }$.

Adams et al. [3] updated Equation (5) by considering the entanglement slippage in the conformational part to capture the strain hardening behavior more accurately. As a result, the total stretch ($\lambda_{i}$) in Equation (5) is replaced by the slippage stretch ($\lambda_{i}^{s}$), which can be obtained by Equation (7):

(7) dln⁡λisdt=Sicγ

where $S_{i}^{c}$ is the deviatoric stresses of the conformational part and γ is the slippage viscosity as shown in Equation (8),

(8) γ=γ01−λmaxnλcritn,

where $\lambda_{max}^{n}$ is the maximum principal network stress,$\;\lambda_{crit}^{n}$ is the critical value of network stretch, and $\gamma_{0}$ is the initial viscosity.

Yan [4] modified Equation (8) by changing $\lambda_{crit}^{n}$ from a constant to a function with respect to temperature and strain rate, as shown in Equation (9):

(9) $$\lambda_{crit}^{n}=-0.0356\times T_{shifted}+15.393$$

where $T_{shifted}$ is the shifted temperature obtained by Equation (10),

(10) $$\log_{10}\left(\alpha \right)=\frac{-0.0111\left(\dot{\varepsilon }-1\right)}{3.627+\dot{\varepsilon }-1}0.9856^{\left(2-2\xi \right)},$$

where α is the shift factor which is the ratio between the shifted temperature and the reference temperature, $\dot{\varepsilon }$ is the strain rate, and ξ is an indicator of deformation mode.

## 4. Hybrid ANN-Based Constitutive Model

### 4.1. Algorithm Selection and Overall Architecture Selection

There are numerous typical algorithms and neural network architectures that can be used to train an ANN-based constitutive model. According to Zhang et al. [34], the most widely used machine learning (ML) algorithm for modeling the stress–strain relationship in the soil domain is a backpropagation neural network (multilayer perceptron). The multilayer perceptron has also been demonstrated in the authors’ previous work to be effectively applicable in displacement-controlled simulations [7,8]. Moreover, due to the particularities of finite element analysis (FEA), integrating a neural network into the simulation of the SBM process in Abaqus requires rewriting the neural network into the VUmat subroutine. The VUmat subroutine has a limited capacity for external variables, and the neural network will be invoked numerous times throughout the simulation process. As a result, it is crucial for the neural network to have a simple structure and involve as few variables as possible to accommodate these constraints effectively. Zhang et al. [34] also proposed that the wide application of the multilayer perceptron was due to its relatively simple structure and strong nonlinear mapping ability, which was also demonstrated by Hagan et al. [35] and Mehrpouya et al. [36]. The feasibility of combining the Multilayer Perceptron with finite element code has already been demonstrated by Kessler et al. [21], Lefik et al. [37], and Hashash et al. [38]. Since the multilayer perceptron has already been applied successfully for modeling nonlinear material behaviors in Abaqus, it is also adopted in the current work.

The aim of the model in the current paper is to predict the nonlinear viscoelastic behavior of PET above T_g_ subjected to large deformation. The initial approach was to try and implement an ANN model only without any constitutive equations. However, whilst this approach worked reasonably well for displacement-controlled simulations as demonstrated in the authors’ previous work [7,8], it was found that the model became unstable when applying it to load-controlled simulations. There was a significant problem in the regions of small strain at the beginning of the analysis where the magnitude of the error for the stress prediction from the ANN was of the same order as that of the magnitude of the stress. To avoid this phenomenon, some physically based equations had to be used in conjunction with the ANN to create a hybrid ANN.

**Figure 11:**
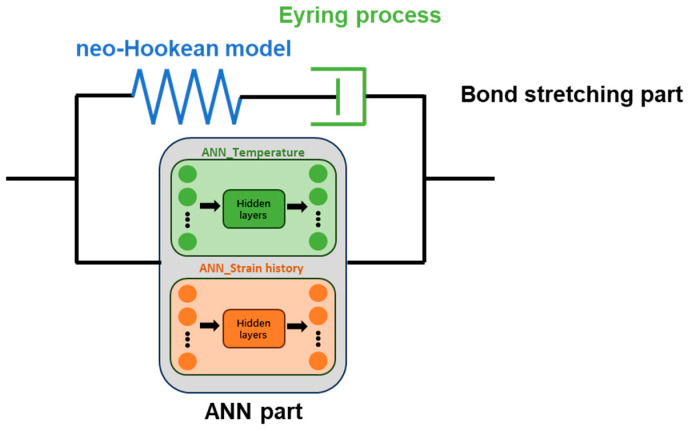
Schematic diagram of hybrid ANN-based model.

Similar concepts of combining ANN models with physical models have already been proposed and demonstrated by several researchers, such as Pandya et al. [14] and Jordan et al. [19]. Pandya et al. proposed [14] an isotropic hardening term k_SV_ obtained from the mixed Swift–Voce law to help the ANN model to take isotropic hardening into account, and Jordan et al. [19] utilized a temperature-dependent Hooke law to help the ANN-based model to describe the elastic behavior. Inspired by these hybrid ANN-based models, the bond stretching part from the Buckley model described by Equations (2)–(4) is retained for a hybrid model in the current work, thus ensuring reasonable predictions in the initial small-strain regime of the model and reducing the effect of error propagations. The architecture of the hybrid ANN model is shown in Figure 11.

As shown in Figure 11, the conformation part from the original Buckley model is replaced by two ANNs, named ‘ANN_Temperature’ and ‘ANN_Strain history’. The ‘ANN_Temperature’ component predicts the conformational stress dependent only on the stretch in the axial and hoop direction, whilst the ‘ANN_Strain history’ will shift this prediction depending on the strain history experienced to date by the element. The details of ‘ANN_Temperature’ and ‘ANN_Strain history’ will be presented in Section 4.3 and Section 4.4.

Despite the wide range of experimental process conditions shown in Table 1, it is inevitable that there will be a range of stretch ratios and strain rates not covered in the training data; in other words, it is impossible to collect all ‘strain history’ data that might occur during simulation process from experiments. This could lead to the ANN model operating in regions where it has not been trained, causing instabilities and errors in the model. In order to deal with this issue, this two-step ANN architecture including ‘ANN_Temperature’ and ‘ANN_Strain history’ was proposed and we adopt this strategy during the practical simulation process so that if an input variable of ‘ANN_Strain history’ exceeds the training range value, it will be limited to the maximum or minimum available from the data and thus ensure that ‘ANN_Temperature’ operates in the region where it has been trained. As mentioned above, ‘ANN_Temperature’ contributes most of the output of this two-step ANN architecture and ‘ANN_Strain history’ only provides shift factors. As a result, this two-step ANN architecture offers superior stability compared to the one-step method.

The ANN part of the model shown in Figure 11 is in parallel with the bond stretching part and operates independently. Therefore, the total stress (σ) can be described by Equation (11):

(11) $$\sigma =\sigma_{b}+\sigma_{ANN}$$

As a result, in order to obtain $\sigma_{ANN}$ (required to train ‘ANN_Temperature’ and ‘ANN_Strain history’), the ‘bond stretching part’ needs to be calculated and subtracted from the total stress, which is equal to the experimental true stress values collected from the free stretch blow experiments (SBM) described in Section 2.

### 4.2. Calculation of Bond Stretching Stress

A detailed procedure of the characterization of the ‘bond stretching part’ can be found in Buckley’s previous work [1,3].

According to the relationship between true strain and true stress at yield points and deformation temperature, the shear (V_s_) and pressure (V_p_) activation volumes can be calculated, which are critical variables in the ‘Eyring process’. Another variable in the bond stretching part is the limiting viscosity (μ_0_), which is temperature-dependent and can be used to predict the temperature effect on the yield stress. Once the limiting viscosity (μ_0_) is defined, the reference viscosity (μ_0_*), limiting temperature (T∞), and viscosity constant (C_v_) can be calculated. By using the characterized values of V_s_, V_p_, μ_0_*, T∞, and C_v_, the equations belonging to the ‘bond stretching part’ are all prepared well. Detailed material constants used in the ‘bond stretching part’ can be found in Appendix A.

**Figure 12:**
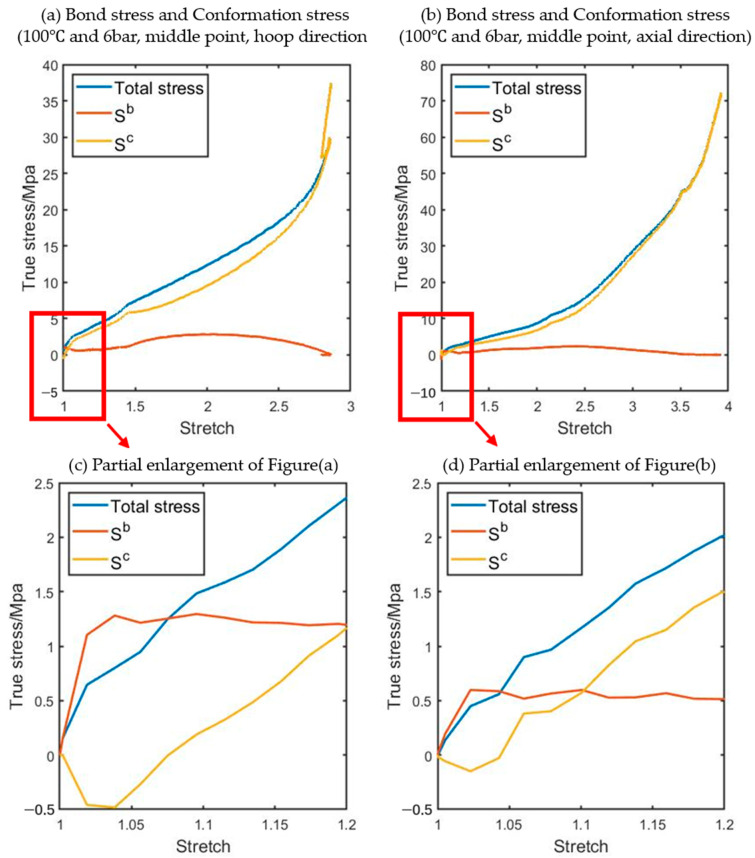
An example of the relationship between S^b^ and S^c^ in both the hoop (**a**) and axial (**b**) direction and partial enlargement views of (**c**,**d**) (the position of the ‘middle point’ is indicated in Figure 2).

An example of the relationship between principal bond stretching deviatoric stress (S^b^) and principle conformational deviatoric stress (S^c^) is shown in Figure 12. This example was obtained from a free stretch blowing experiment conducted at 100 °C with a supply pressure of 8 bar (named P8N6T100 in Table 1).

According to Figure 12c,d, it can be observed that S^b^ contributes significantly to the total stress at the beginning of deformation, particularly when the stretch is smaller than 1.02. However, with the development of stretching, the value of S^b^ decreases rapidly and approaches zero as the stretch increases and is subsequently dominated by the conformational part, as shown in Figure 12a,b. In the current work, the conformational part will be replaced by the ANNs.

In conclusion, although the ‘bond stretching part’ exists in the hybrid ANN-based constitutive model shown in Figure 11, it only contributes to the stress at the beginning of the SBM process where the strains are small, and it operates independently from the ‘ANN part’.

### 4.3. ANN_Temperature

**Figure 13:**
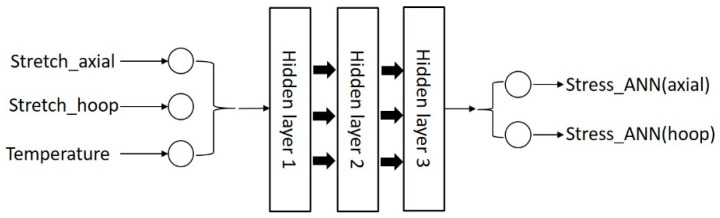
The architecture of ‘ANN_Temperature’.

The architecture of ‘ANN_Temperature’ is shown in Figure 13. The number of layers and the number of neurons in each layer will be introduced in the next section (Section 5).

The inputs to the model are chosen to reflect the most important parameters that influence the behavior of the material. ‘Stretch_axial’ and ‘Stretch_hoop’ represent the principal stretches in the axial and hoop direction, respectively, whilst ‘Stress_ANN(axial)’ and ‘Stress_ANN(hoop)’ are the stresses predicted by ‘ANN_Temperature’ in the axial and hoop direction, respectively. Our previous work [7,8] has already demonstrated that one multilayer perceptron is able to accurately describe the relationship between strain, stress, and other variables; therefore, ‘ANN_Temperature’ also adopts a conventional architecture of a multilayer perceptron. The detailed transfer functions, weight matrices, and bias vector definitions were also explained in our previous work [7,8]. Note that strain rate is not an input parameter to this ‘ANN_Temperature’ component; so, the prediction from ‘ANN_Temperature’ is independent of this variable and is therefore the best fit prediction at the given temperature across all of the experiments conducted at different strain rates.

### 4.4. ANN_Strain History

**Figure 14:**
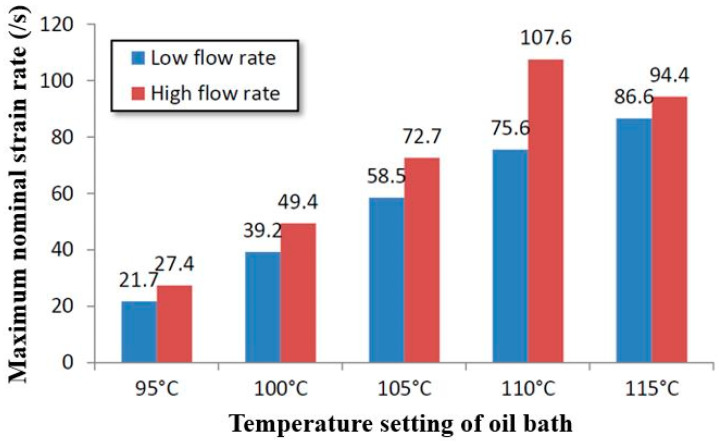
The effect of mass flow rate and temperature on the maximum strain rate.

It has been demonstrated experimentally that strain rate has an important influence on the behavior of PET above T_g_ during biaxial tension experiments [1,3]. However, the influence of strain rate decreases in the SBM process. The strain rate during the SBM process is mainly influenced by the mass flow rate of air and the temperature of the preform. The maximum strain rate appearing in every free stretch blow test in Table 1 was recorded and is shown in Figure 14.

The ‘low flow rate’ in Figure 14 represents ‘flow index 2’ shown in Table 1 and the high flow rate represents ‘flow index 6’. According to Yan [4], the strain rate mainly influences the behavior of PET during SBM through the ‘self-heating’ phenomenon, and Yan also proposed an empirical equation to quantify the influence produced by a high strain rate.

**Figure 15:**
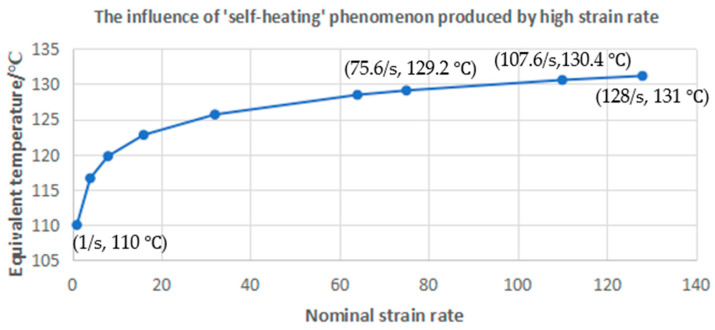
The temperature increase produced by the high strain rate during the SBM process.

As shown in Figure 14, the difference in the maximum strain rate is most obvious when the oil bath temperature is 110 °C; so, the ‘110 °C scenario’ is also adopted in the current work as an example. Yan [4] proposed a concept called ‘equivalent temperature’ in his previous research which is able to quantitatively transfer the influence produced by a high strain rate to the effects provided by temperature. As shown in Figure 15, according to Yan’s empirical equation, the behavior of PET (strain–stress curve) stretched at a nominal strain rate of 128/s and at 110 °C is similar to the behavior of PET (strain–stress curve) stretched at a nominal strain rate of 1/s and at 131 °C. It can therefore be observed that the influence of strain rate on PET decreases with the increase in strain rate. The equivalent temperatures for strain rates of 75.6/s and 107.6/s are 129.2 °C and 130.4 °C—a small difference with little impact on stress strain behaviour and hence can be ignored. As result, compared with the biaxial stretching experiments, the strain rate is not as important in the SBM process since it mainly operates in the high-strain-rate range even when the ‘flow index’ is et to 2. As a result, the influence of strain is removed from the main ANN ( ‘ANN_Temperature’).

Gorji et al. [39] proposed an equation when developing a neural network model for large deformation of metals which is shown in Equation (12). This equation will be used in the ‘ANN_Strain history’ component of the model in this work. The equation effectively captures the stretch experienced by the material ($\varepsilon \left(t\right))$ at a specific instance in time (τ).

(12) $$\bar{\varepsilon }\left(\tau \right)=\frac{1}{\tau }\int_{0}^{\tau }\varepsilon \left(t\right)dt$$

According to Gorji et al. [39], due to the integration operation, the variable ($\bar{\varepsilon }\left(\tau \right)$) obtained in Equation (12) is also able to capture the strain path experienced by the element during the simulation; so, this variable is named ‘strain history’ in this paper and it is much more stable than directly using the stretch at a given time as it is less susceptible to fluctuations. Integrating this variable into the ANN model provides an additional benefit by helping it incorporate historical variations in strain (ε). This reduces oscillations in predictions and enhances stability. Since the stretch is the primary input for the ‘ANN_Temperature’ model (as shown in Figure 13), considering its past values allows the model to make more accurate predictions. This advantage allows the current hybrid ANN-based constitutive model to avoid exhibiting strong oscillations, which is a typical disadvantage of a multilayer perceptron.

**Figure 16:**
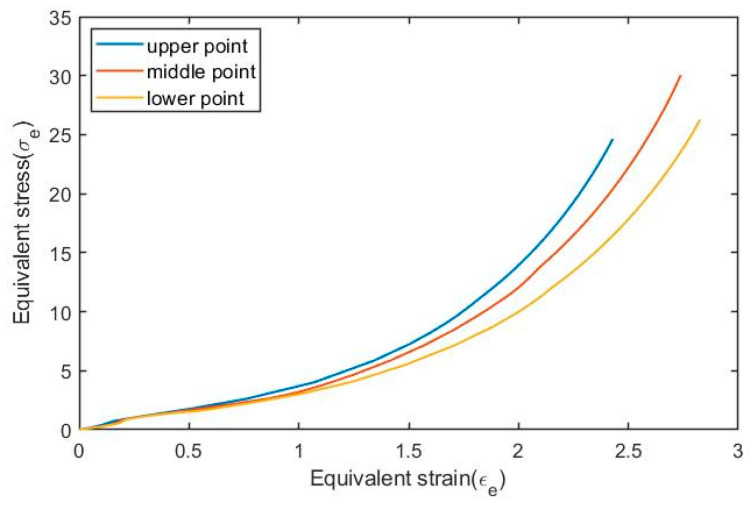
Equivalent stress–equivalent strain curves of selected points in P8N6T115.

As shown in Figure 2, three different sampling points are selected on the wall of the preform, named the upper point, middle point, and lower point. During experiments, stress and strain data in both axial and hoop directions were collected for these three sampling points. To combine the data from both directions (axial and hoop direction), the concepts of equivalent stress and equivalent strain (von Mises strain) are adopted in the current paper. The relationships between the equivalent stress and equivalent strain of these selected sampling points are shown in Figure 16.

The equivalent sterss $\sigma_{e}$ and equivalent strain ($\varepsilon_{e}$) shown in Figure 16 are obtained by using Equations (13) and (14), respectively:

(13) $$\sigma_{e}=\sqrt{\frac{{\left(\sigma_{1}-\sigma_{2}\right)}^{2}+{\left(\sigma_{2}-\sigma_{3}\right)}^{2}+{\left(\sigma_{3}-\sigma_{1}\right)}^{2}}{2}}$$

(14) $$\varepsilon_{e}=\sqrt{\frac{{\left(\varepsilon_{1}-\varepsilon_{2}\right)}^{2}+{\left(\varepsilon_{2}-\varepsilon_{3}\right)}^{2}+{\left(\varepsilon_{3}-\varepsilon_{1}\right)}^{2}}{2}}$$

According to Figure 16, it can be observed that the behavior of PET varies depending on the location of the selected element on the preform even in the same free stretch experiment because a different position means that these three sampling points have undergone different strain histories.

**Figure 17:**
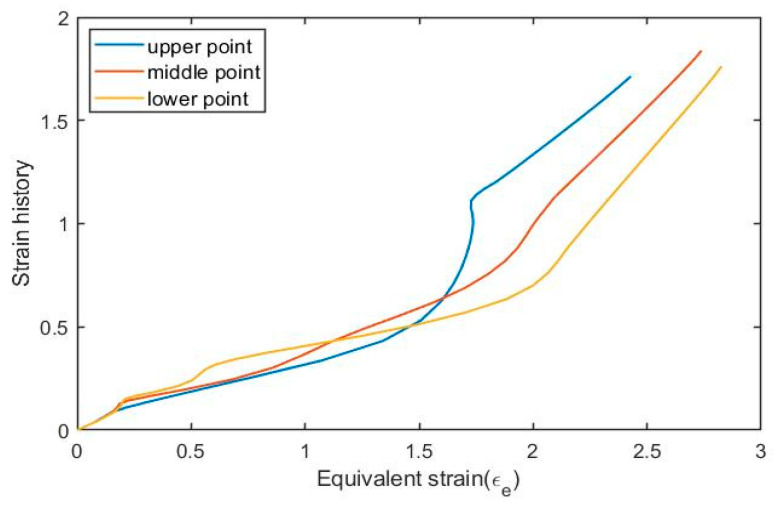
The development of the strain history of selected points in P8N2T115.

The development of the ‘strain history’ (obtained by Equation (12)) of these selected sampling points vs. equivalent strain is shown Figure 17. When comparing Figure 17 with the stress–strain behavior in Figure 16, it can be observed that the ‘strain history’ is a suitable input variable to be used in an ANN-based constitutive model since (i) it has more direct correlation with the stress–strain curve and (ii) it is has a stable evolution.

**Figure 18:**
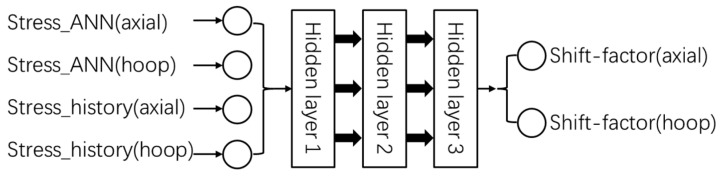
The structure of ‘ANN_Strain history’.

The structure of ‘ANN_Strain history’ is shown in Figure 18. The number of layers and the number of neurons in each layer will be introduced in the next section (Section 5).

‘Stress_ANN(axial)’ and ‘Stress_ANN(hoop)’ are inherited from ‘ANN_Temperature’ and they share the same values. As mentioned above, ‘Strain_history(axial)’ and ‘Strain_history(hoop)’ represent the ‘strain history’ of the axial and hoop directions separately which are calculated by Equation (12). ‘Shift-factor(axial)’ and ‘Shift-factor(hoop)’ are factors required to shift the prediction of stress from ‘ANN_Temperature’, allowing for the prediction of the effect of strain history.

**Figure 19:**
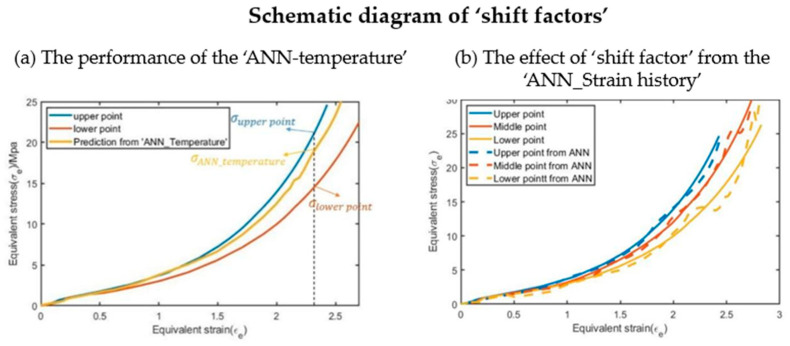
Schematic diagram of ‘shift factors’ for selected points in P8N6T115.

An example is shown in Figure 19a where the outputs of ‘ANN_Temperature’ for the upper point and the lower point of the preform are both the same. This is shown by the yellow curve and occurs because they share the same temperature (locations of ‘upper’ and ‘lower point’ can be found in Figure 2). With the help of the ‘ANN_Strain history’ calculation of the shift factor, the equivalent stress–equivalent strain curves for the upper point and the lower point are separated because the ‘strain history’ of these two points are different, resulting in different shift factors, as shown in Figure 19b. The concept of tuning the output of the ANN with extra factors was inspired from the work of Pandya et al. [14], who applied an isotropic hardening term k_SV_ obtained from the mixed Swift–Voce law to adjust the result from the ANN to reflect the influence of isotropic hardening. In other words, ‘ANN_Strain history’ is fine-tuning the outputs from ‘ANN_Temperature’ to take the influence of strain history into account.

### 4.5. A Walk-Through of the Hybrid ANN-Based Constitutive Model

**Figure 20:**
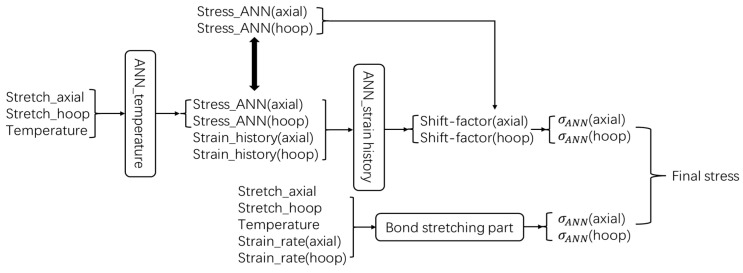
A flow chart demonstrating the calculation procedure of the hybrid ANN-based constitutive model.

The hybrid ANN-based constitutive model is composed of three components. These include the physical equations governing the bond stretching part, elaborated in Section 4.2, ‘ANN_Temperature’ discussed in Section 4.3, and ‘ANN_Strain history’ presented in Section 4.4. A detailed flow chart illustrating the integration of these three components to determine the stress output of the hybrid ANN-based constitutive model is depicted in Figure 20.

The entire constitutive model can be separated into two parts, the bond stretching part and the ANN part. An explanation of the ‘bond stretching part’ can be found in Buckley’s previous work [1,3]. As a result, the detailed calculation procedures of this part is not included in the current paper. According to Figure 20, in order to obtain ‘Stress_ANN(axial)’ and ‘Stress_ANN(hoop)’, ‘Stretch_axial’ and ‘Stretch_hoop’ are firstly input into ‘ANN_Temperature’ introduced in Section 4.3. It is worth noting that ‘Stress_ANN(axial)’ and ‘Stress_ANN(hoop)’ are not only input variables of ‘ANN_Strain history’ introduced in Section 4.4 but are also used in the equation for calculating the ‘Final stresses’. As explained in Section 4.4, in order to take the effect of strain history into account, ‘Shift-factor(axial)’ and ‘Shift-factor(hoop)’ need to be multiplied with the prediction of stress from ‘ANN_Temperature’ (‘Stress_ANN(axial)’ and ‘Stress_ANN(hoop)’). The ANN part and the ‘bond stretching part’ are added together as defined in Equation (11) to obtain the final stress of the hybrid ANN-based constitutive model.

The entire constitutive model can be separated into two parts, the bond stretching part and the ANN part. An explanation of the ‘bond stretching part’ can be found in Buckley’s previous work [1,3]. As a result, the detailed calculation procedures of this part is not included in the current paper. According to Figure 20, in order to obtain ‘Stress_ANN(axial)’ and ‘Stress_ANN(hoop)’, ‘Stretch_axial’ and ‘Stretch_hoop’ are firstly input into ‘ANN_Temperature’ introduced in Section 4.3. It is worth noting that ‘Stress_ANN(axial)’ and ‘Stress_ANN(hoop)’ are not only input variables of ‘ANN_Strain history’ introduced in Section 4.4 but are also used in the equation for calculating the ‘Final stresses’. As explained in Section 4.4, in order to take the effect of strain history into account, ‘Shift-factor(axial)’ and ‘Shift-factor(hoop)’ need to be multiplied with the prediction of stress from ‘ANN_Temperature’ (‘Stress_ANN(axial)’ and ‘Stress_ANN(hoop)’). The ANN part and the ‘bond stretching part’ are added together as defined in Equation (11) to obtain the final stress of the hybrid ANN-based constitutive model.

## 5. Training the ANN Part of the Constitutive Model

It is worth noting that the experimental data of P8N2T110 and P8N2T115 shown in Table 1 have already been removed from the experimental database before the training procedure because they are used to validate the performance of the hybrid ANN-based constitutive model in Abaqus.

As mentioned in Section 2.2, each experiment shown in Table 1 is able to provide 85 strain–stress curves. Since the data sets for P8N2T110 and P8N2T115 have been removed, the experimental database now comprises data from eight experiments. Consequently, the database contains a total of 680 strain–stress curves. In line with work conducted by Hagan et al. [35], 85% of these strain–stress curves are selected for training ‘ANN_Temperature’ and ‘ANN_Strain history’. As a result, for each experiment, eight complete strain–stress curves are classified into the testing database and the others belong to the training database. For these eight curves, six curves are selected randomly and the complete strain–stress curves of the upper point and the lower point are included in the testing database.

### 5.1. Detailed Training Settings

The neural network was initially built, trained, and validated using the MATLAB R2023b neural network toolbox.

‘ANN_Temperature’ shown in Figure 13 is a typical multilayer perceptron. A similar ANN architecture with three layers was adopted by Tao et al. [40]. They utilized this ANN to establish a relationship between in-plane strains and the corresponding stiffness matrix in an UMAT subroutine in Abaqus. Since the number of input and output variables, the size of the experimental database, and the complexity of the function are similar, the number of layers used by Tao was also used in this model.

Le et al. [41] proposed that an ANN should use as few neurons as possible on the premise of ensuring accuracy during training, and they demonstrated that the performance surface of an ANN with a simpler architecture is smooth and monotonic and thus is more suitable to describe a physical phenomenon. In order to avoid overfitting and an unrealistic performance surface with localized, inconsistent fluctuations, the number of neurons in each layer was set at nine, a relatively small value that was determined after trial-and-error testing. The ‘ANN_Temperature’ architecture is defined by the nomenclature [3,9,9,9,2], which indicates that the ANN has three input variables, two output variables, and three hidden layers, with each layer having nine neurons. The training epoch was set as 1000.

The details on training algorithm selection (Bayesian regularization backpropagation algorithm) and trial-and-error testing for ‘ANN_Temperature’ can be found in our previous work [7,8]. A detailed work flow on how to train an ANN by using experimental data sets from the materials domain is described by Pal et al. [42], whilst information about the training algorithm and regularization have already been explained by Kim et al. [43].

The training program and algorithm used by ‘ANN_Strain history’ are the same as those used by ‘ANN_Temperature’ described above. After the same trial-and-error test, the structure of ‘ANN_Strain history’ was defined as [4,4,5,5,2], meaning the ANN has four input variables, two output variables, and three hidden layers of four, five, and five neurons, respectively.

It is worth noting that all training data were normalized before training. The magnitude of all training data was scaled equally into the interval of (−1,1) and re-normalization on their outputs was also necessary. The ‘normalization’ and the ‘re-normalization’ procedures were controlled by the algorithm automatically.

### 5.2. Validation in Testing Database

Once both ‘ANN_Temperature’ and ‘ANN_Strain history’ are trained, the entire ‘ANN part’ (‘ANN_Temperature’ with the help of ‘ANN_Strain history’, as shown in Figure 11) was validated via the testing database to verify the accuracy of the training procedure. This operation was implemented in MATLAB. As mentioned above, the complete strain–stress curves of the upper point and the lower point were included in the testing database. Therefore, the performance of the entire ‘ANN part’ when simulating PET behavior at the ‘upper point’ and the ‘lower point’ position is shown in this section.

**Figure 21:**
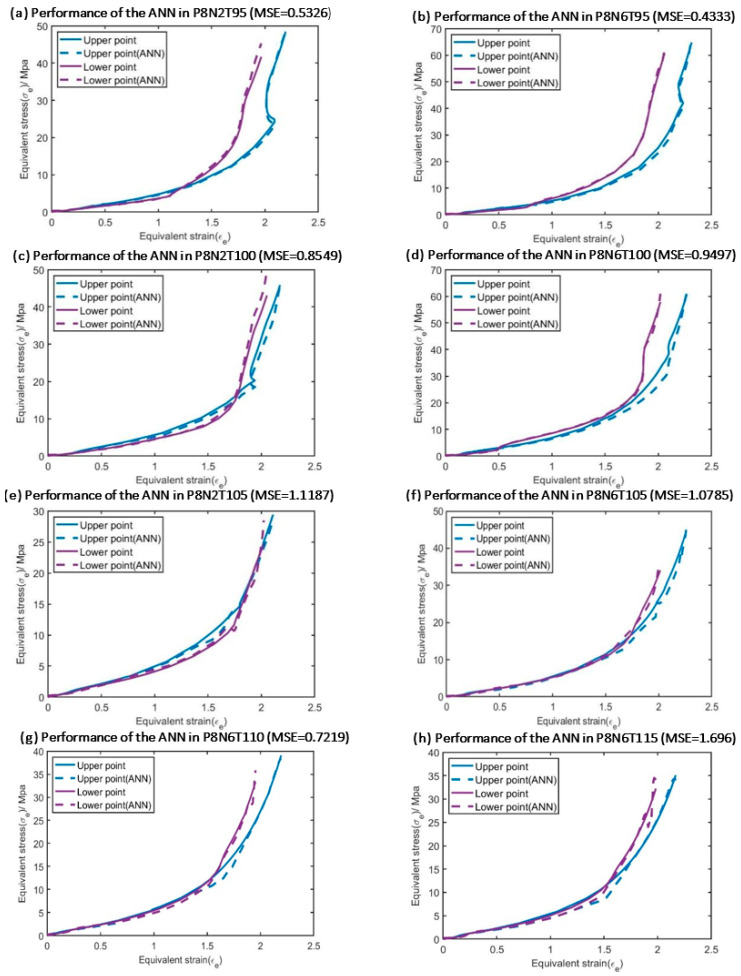
The testing results of the hybrid ANN-based constitutive model for the testing database from Table 1 on the upper and lower points of the preform.

In order to quantify the accuracy of the ANN models, the results shown in Figure 21 were evaluated by both the Mean Square Error function (MSE) and the Mean Relative Error (MRE), which are obtained by Equations (15) and (16), respectively.

(15) $$MSE=\frac{\sum {\left(\sigma_{exp}-\sigma_{sim}\right)}^{2}}{n}$$

(16) $$MRE=\frac{\sum \left|\frac{\sigma_{exp}-\sigma_{sim}}{\sigma_{exp}}*100\%\right|}{n}$$

$\sigma_{sim}$ is the simulation stress value of each data point, $\sigma_{exp}$ is the corresponding experimental data, and n is the total number of data points.

According to Figure 21 and Table 2, ‘ANN_Temperature’ and ‘ANN_Strain history’ have accurate performance when they are used to predict the stress output of data in the testing database. As a result, the training process of both ‘ANN_Temperature’ and ‘ANN_Strain history’ can be considered accurate enough for the current work.

## 6. Validation and Comparison

### 6.1. Implementation in Abaqus

The hybrid ANN-based model was implemented in Abaqus by a VUMAT subroutine. PET’s material properties were defined by density (1.33 g/cm^3^) and Poisson’s ratio (0.495).

**Figure 22:**
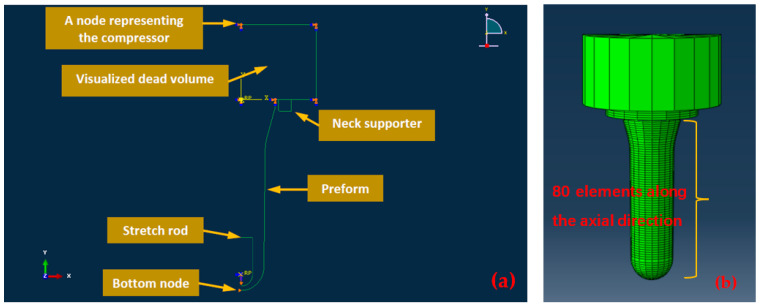
The detailed FE model used for the free stretch blow simulation. (**a**) The annotated CAE interface to illustrate the model of the free stretch blow process in ABAQUS/Explicit; (**b**) the FE preform model with 80 SAX1 elements.

When the ratio between the radius and wall thickness of a cylindrical object is approximately 1/10, thin-walled pressure vessel theory can be applied, allowing the stress through the thickness to be neglected [24]. Given the radius-to-thickness ratio in a typical bottle, this theory was adopted, and the axisymmetric preform was modeled using shell elements (SAX1 element type) in Abaqus. The SAX1 element is particularly well suited for analyzing structures that exhibit rotational symmetry, such as pressure vessels, pipes, and rotating shells. It is designed to accurately simulate the distribution of stress and strain within these structures, which is essential for predicting their mechanical response to applied loads. This element is capable of accounting for both the hoop (circumferential) and axial stresses that arise in thin-walled pressure vessels when they are subjected to internal pressure. The preform as shown in Figure 2 has been meshed with 80 SAX1 elements, with each element assigned a corresponding thickness. The preform is constrained in the axial direction at the position just after the neck support ring and is constrained in the axial direction at the preform tip. Similar to Nixon et al. [44], a fluid cavity is set up inside the preform to model the different mass flow rates of air entering the preform for each experiment, with a fluid exchange property applied to represent the flow of air from the compressor to the preform. Non-deformable items such as the stretch rod and preform clamp were modeled using rigid elements (RAX2). The detailed model of the free stretch blow process used in Abaqus is shown in Figure 22.

**Figure 23:**
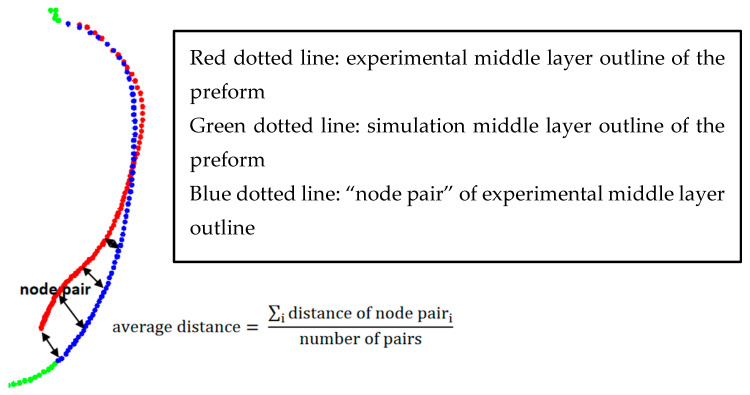
Illustration of distance between node pair, and equation of calculating average distance.

The hybrid ANN-based constitutive model in the current paper is developed for simulating the SBM process specifically. Compared with the strain–stress curves of PET at some specific positions, it is also important to quantify the accuracy of the final geometry of the preform predicted by the hybrid ANN constitutive model during the free blow experiments since it has a direct relationship with the final product of the SBM process. In order to quantitatively compare these preforms’ geometries predicted by the Buckley model and the hybrid ANN model in the following section, a concept called ‘average nodal distance’ was proposed. A schematic diagram and calculation equation are shown in Figure 23. As shown in Figure 23, the middle layer outline of the preform collected from the experiment is represented by the red dotted line, while the corresponding simulation result is shown by the green dotted lines. Figure 3c indicates the relative positional relationship between high-speed cameras and the preform during experiments. These two cameras were placed horizontally at the middle of the preforms during the experiments so they could not capture the evolution of strain for these positions located at the top and bottom of the preforms due to the viewing angle. This is the reason why the middle layer outline (red solid line) is missing at the bottom of Figure 23. In other words, the ‘strain’ variable for some nodes in the simulation model does not have corresponding experimental data. These nodes which have experimental ‘strain’ data collected by these two high-speed cameras are shown by the blue dotted line in Figure 23. The two nodes which share the same position in the simulation model and the preform sample at the beginning of the free stretch blow process are called one ‘node pair.’ The variable ‘average distance’ is obtained from each ‘node pair’.

### 6.2. Free Blow Validation

As mentioned above, the experimental data of P8N2T110 and P8N2T115 have been removed from the training database. Therefore, test conditions of P8N2T110 and P8N2T115 are used to validate the performance of the hybrid ANN-based constitutive model in Abaqus/Explicit in the current section.

**Figure 24:**
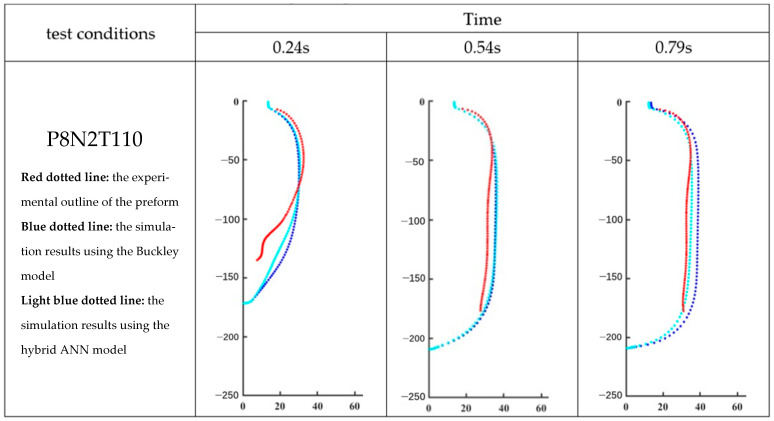
Shape comparison of P8N2T110 in MATLAB.

**Figure 25:**
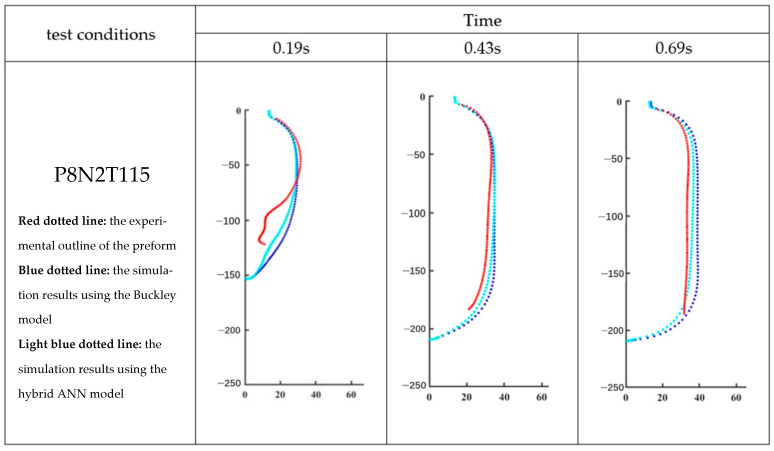
Shape comparison of P8N2T115 in MATLAB.

According to Section 4, the hybrid ANN-based model is developed from the Buckley model. Therefore, the evolution of the preform as predicted by the hybrid ANN-based constitutive model and the Buckley model developed by Yan [4] at different time moments is shown in Figure 24 and Figure 25, respectively. MATLAB is used to plot these geometries by using coordinate data of all elements which are exported from Abaqus/Explicit. The outline of the preform captured by the DIC analysis during the free stretch blow experiment is represented by the red dotted line, while the simulation results by the Buckley model and the hybrid ANN-based model are shown, respectively, by the blue and light blue dotted lines.

In order to show the whole process clearly, each entire free stretch blow simulation is represented by three pictures which are recorded at different time points, and these three diagrams divide this process as equally as possible; for example, the complete simulation for P8N2T110 lasts 0.79 s, so this process is represented by the figures recorded at 0.24 s, 0.54 s, and 0.79 s.

**Figure 26:**
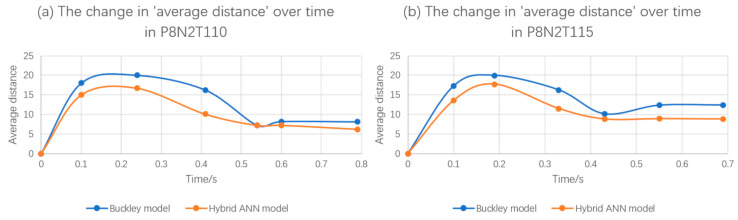
‘Average distance’ comparison between the Buckley model and the hybrid ANN model for (**a**) P8N2T110 and (**b**) P8N2T115.

The values of ‘average distance’ of all simulations predicted by the Buckley model and the hybrid ANN-based model are shown in Table 3. Based on Figure 24 and Figure 25, Figure 26 presents the variation in the average distance over time for the simulation process of experiments P8N2T110 and P8N2T115.

According to Figure 26, the hybrid ANN-based constitutive model has already demonstrated that it is able to be stably implemented in Abaqus/Explicit via the VUMAT subroutine under load-controlled scenarios and can predict preform geometry during the free stretch blow process with relatively high accuracy compared with the Buckley model.

## 7. Discussion

Although a hybrid ANN-based constitutive model has been successfully implemented in a free stretch blow simulation and validated against experimental data, there is still potential to explore the limits of the model beyond those described in this paper so that we can verify the robustness of the model. The validation in the free stretch blow process for example could be expanded to include experiments using preforms of the same material but with different geometries and under different process conditions. In addition, experiments using more extreme conditions could present a challenge to the model with its current architecture. It has been reported in the literature that there is an influence of the sequential mode of deformation, i.e., a strain history involving a significant stretch in one direction followed by a subsequent stretch in the other direction is able to influence the evolution of the microstructure and the resulting stress–strain behavior. With the current approach of taking strain history into account, it is unlikely that the model will be able to capture significant changes in the stress–strain behavior produced by this mode of deformation. Whilst strain history is a good starting point, it is likely that a more complex function that takes account of the strain history of the polymer and the strain path experienced to arrive at the current state will be required.

The prediction by the ANN whilst good still has some limitations; for example, in Figure 19, the prediction for the lower point on the preform displays a fluctuation in the stress–strain curve rather than a smooth monotonic stress–strain curve observed experimentally. This is likely a result of the overfitting phenomenon that can be typically found when training ANNs. Although lots of methods have already opted to decrease the level of oscillations in ‘ANN_Temperature’ and ‘ANN_Strain history separately, for example,’ANN_Strain history’ has already been designed as a kind of dynamic model, the oscillation level of the output of the ANN-based constitutive model is still slightly larger than the corresponding oscillation level in a single ANN due to the relationship between the outputs of these two ANNs (they need to be multiplied by each other). It is unclear at present what influence this will have on the free stretch blow simulations but it can likely be minimized through further optimization.

The combination of ‘ANN_Temperature’ and ‘ANN_Strain history’ may increase the oscillation level of the ANN model’s output. However, it significantly enhances the model’s stability under conditions beyond the training range or with extreme strain histories compared to a single ANN model. As depicted in Figure 13, the input variables for ‘ANN_temperature’ encompass ‘stretch_axial’, ‘stretch_hoop’, and temperature. A comprehensive database comprising these variables can be readily obtained through planar tensile testing. Consequently, the likelihood of the input data for ‘ANN_Temperature’ exceeding the training set during the stretch blow molding process is minimal. The input of ‘ANN_Strain history’ includes ‘strain history’, which may cause its input data to exceed the training set. However, as illustrated in Figure 20, the output of ‘ANN_Strain history’ functions as a shift factor to fine-tune the results of ‘ANN_temperature’. Therefore, when the input strain history of ‘ANN_Strain history’ exceeds the training set, its output can be confined within a rational and relatively small range. According to experimental data, this range is [0.76, 1.78] in this study. In conclusion, the current structure ensures the stability of ‘ANN_Temperature’ and its substantial contribution to the final output values. ‘ANN_Strain history’ is designed to improve the accuracy of the output from ‘ANN_Temperature’, but it may encounter data exceeding the training set. When such a situation arises, the role of ‘ANN_Strain history’ is restricted. A strategy that sacrifices a certain degree of accuracy is adopted to maintain overall stability.

Despite the advantage described above, the experimental approach combined with the hybrid ANN-based constitutive model offers other advantages over the traditional testing methods and physical-based constitutive modeling approach. These include the ability to automatically produce a fitted material model for any material that can be stretch blow molded much more efficiently (days vs. weeks).

As outlined in Section 4 and Section 5, the training sets for ‘ANN_Temperature’ and ‘ANN_Strain history’ comprise numerous stress–strain curves acquired under fitting experimental conditions. To adapt these models to other viscoelastic polymers, only the corresponding stress–strain curves of those polymers need to be prepared, with no changes to the models’ architectures. Thus, ‘ANN_Temperature’ and ‘ANN_Strain history’ exhibit high versatility for different polymers. This advantage is particularly important for the stretch blow molding process given the huge interest in replacing petroleum-based polymers such as PET with bio-based materials such as polyethylene furanoate (PEF) and polyhydroxyalkanoates (PHAs). Whilst it would have previously taken months to study and test these polymers so that we could build new constitutive laws and establish new processing and design rules to account for their different properties, there is now the potential for this to be achieved in weeks with the combination of the semi-automatic test rig, automated training via the ANN, and the stretch blow molding simulation incorporating the ANN.

## 8. Conclusions

1. A new semi-automatic experimental rig has been developed enabling a rich data set of stress–strain curves (850 strain–stress curves) directly from preforms under conditions representative of the stretch blow molding process.
2. A hybrid ANN combining an Eyring function proposed by Buckley et al. for capturing the small-strain behavior in parallel with an ANN model for capturing the temperature-dependent large-strain nonlinear viscoelastic behavior of PET has been developed and validated over a range of conditions typically used in the stretch blow molding process.
3. The ANN has demonstrated the ability to be utilized in a simulation of stretch blow molding (without mold), thus validating its stability in a load-controlled scenario and its ability to predict the blowing behavior of a preform.

## Figures and Tables

**Table 1:** Summary of experiments conducted.

Experiment Label (Press/Flow/Temp)	Pressure, P (bar)	Flow Index, N (1–6)	Temperature Setting of Oil Bath (°C)
P8N2T95	8	2	95
P8N6T95	8	6	95
P8N2T100	8	2	100
P8N6T100	8	6	100
P8N2T105	8	2	105
P8N6T105	8	6	105
P8N2T110	8	2	110
P8N6T110	8	6	110
P8N2T115	8	2	115
P8N6T115	8	6	115

**Table 2:** The Mean Relative Error (MRE) of the experiments shown in Figure 21.

Experiment Label	Mean Relative Error
P8N2T95	1.37%
P8N6T95	1.16%
P8N2T100	2.14%
P8N6T100	2.42%
P8N2T105	2.57%
P8N6T110	2.00%
P8N6T115	4.14%

**Table 3:** Detailed ‘average distance’ of simulation results at finial moment.

	Buckley Model Simulation	Hybrid ANN-Based Constitutive Model Simulation
P8N2T110	8.11	6.21
P8N2T115	12.39	8.83

## Data Availability

Data are contained within the article.

## Appendix A

**Table A1:** Material constants of ‘bond stretching part’.

Bond stretching part	shear activation volume V_s_ (m^3^ mol^−1^)	2.814 × 10^−3^
	pressure activation volume V_p_ (m^3^ mol^−1^)	0.526 × 10^−3^
	reference viscosity $\mu_{0}^{*}$ (Mpa)	1.8165
	limiting temperature $T_{\infty }$ (K)	342.61
	viscosity constant C^v^ (K)	56.09
